# Green synthesis of biogenetic Te(0) nanoparticles by high tellurite tolerance fungus *Mortierella* sp. AB1 with antibacterial activity

**DOI:** 10.3389/fmicb.2022.1020179

**Published:** 2022-10-05

**Authors:** Bo Ao, Fei He, Jing Lv, Junming Tu, Zheng Tan, Honglin Jiang, Xiaoshan Shi, Jingjing Li, Jianjun Hou, Yuanliang Hu, Xian Xia

**Affiliations:** ^1^Hubei Key Laboratory of Edible Wild Plants Conservation and Utilization, Huangshi Key Laboratory of Lake Environmental Protection and Sustainable Utilization of Resources, Hubei Engineering Research Center of Characteristic Wild Vegetable Breeding and Comprehensive Utilization Technology, Hubei Normal University, Huangshi, China; ^2^Hubei Provincial Center for Disease Control and Prevention, Wuhan, China; ^3^Department of Virology, University of Helsinki, Helsinki, Finland

**Keywords:** *Mortierella*, Te(IV) reduction, Bio-TeNPs, antibacterial, cytotoxicity

## Abstract

Tellurite [Te(IV)] is a high-toxicity metalloid. In this study, a fungus with high Te(IV) resistance was isolated. Strain AB1 could efficiently reduce highly toxic Te(IV) to less toxic Te(0). The reduced products formed rod-shaped biogenetic Te(0) nanoparticles (Bio-TeNPs) intracellularly. Further TEM-element mapping, FTIR, and XPS analysis showed that the extracted Bio-TeNPs ranged from 100 to 500 nm and consisted of Te(0), proteins, lipids, aromatic compounds, and carbohydrates. Moreover, Bio-TeNPs exhibited excellent antibacterial ability against *Shigella dysenteriae*, *Escherichia coli*, *Enterobacter sakazakii*, and *Salmonella typhimurium* according to inhibition zone tests. Further growth and live/dead staining experiments showed that *E*. *coli* and *S*. *typhimurium* were significantly inhibited by Bio-TeNPs, and cells were broken or shriveled after treatment with Bio-TeNPs based on SEM observation. Additionally, the antioxidant and cytotoxicity tests showed that the Bio-TeNPs exhibited excellent antioxidant capacity with no cytotoxicity. All these results suggested that strain AB1 showed great potential in bioremediation and Bio-TeNPs were excellent antibacterial nanomaterials with no cytotoxicity.

## Introduction

Tellurium (Te) is a semimetallic element belonging to the chalcogen family. It has been used in metallurgy, inorganic and organic chemistry, materials science, and especially in the electronics industry (Chivers and Laitinen, 2015). Its wide application makes it an emerging pollutant in the environment (Wiklund et al., 2018; Filella et al., 2019), and it can accumulate in foods (Doulgeridou et al., 2020). Te compounds are available in various oxidation states (−II, 0, +II, +IV, and +VI) (Vavrova et al., 2021). Te exists mainly in the form of Te(IV) and Te(VI) in the natural environment (Belzile and Chen, 2015). Among various Te compounds, Te(IV) is the most toxic (Gad and Pham, 2014). Although there are few studies on the human toxicity of tellurium, Te(IV) has been shown to cause metallic taste, nausea, and vomiting (Yarema and Curry, 2005). The toxicity of Te(IV) is greater than that of cadmium, mercury, chromium, and copper in some microorganisms (Chasteen et al., 2009). Te(0) has less or no toxicity compared to Te(IV) (Gad and Pham, 2014). Accordingly, the reduction of highly toxic Te(IV) to less or no toxic Te(0) is of great significance for environmental pollution remediation and human health (Fawzy, 2019). Microorganisms play an important role in the Te biochemical cycle in the earth and show great potential in Te(IV) contamination bioremediation (Missen et al., 2020).

Microbes have developed multiple mechanisms to cope with the high toxicity of Te(IV), including (1) decreased influx, (2) efflux, (3) reduction, and (4) methylation (Chasteen et al., 2009; Vavrova et al., 2021). Reduction is one of the most important detoxification mechanisms of microbes. In recent years, dozens of Te(IV)-reducing bacteria have been found, such as *Escherichia coli* (Wang et al., 2011), *Shewanella oneidensis* (Kim et al., 2013), *Pseudomonas* sp. (Pugin et al., 2014), *Rhodobacter capsulatus* (Borghese et al., 2016), *Rhodococcus aetherivorans* (Presentato et al., 2016), *Erythromonas ursincola* (Maltman et al., 2017), *Bacillus selenitireducens* (Wang et al., 2019), *Raoultella* sp. (Nguyen et al., 2019), *Shinella* sp. (Wu et al., 2019), and *Aeromonas hydrophila* (Castro et al., 2020). However, the Te(IV) resistance of reported Te(IV)-reducing bacteria ranges from 0.01 to 8 mM, which indicates low-level resistance, possibly limiting their application (Maltman and Yurkov, 2019; Wu et al., 2019). Usually, fungi can grow in high concentrations of heavy metals compared to bacteria (Zambonino et al., 2021). Currently, several fungi, *Phanerochaete chrysosporium* (Espinosa-Ortiz et al., 2017), *Aspergillus welwitschiae* (Abo Elsoud et al., 2018), *Penicillium chrysogenum* (Barabadi et al., 2018), *Aureobasidium pullulans*, *Mortierella humilis*, *Trichoderma harzianum* (Liang et al., 2019), and *Phoma glomerata* (Liang et al., 2020), have been found to have the ability to reduce Te(IV). However, those fungal studies mainly focused on tellurium nanoparticle production. The application of the Te(IV) reducing fungi and the fungal Bio-TeNPs still need to be further studied.

Generally, the microbial Te(IV) reduction products that have been identified are Te(0) nanoparticles (Zambonino et al., 2021). Currently, the application studies based on biogenetic Te(0) nanoparticles (Bio-TeNPs) have primarily focused on bacterial Bio-TeNPs. Some bacterially produced Bio-TeNPs possess antimicrobial, antioxidant, and photocatalysis properties (Zare et al., 2012; Shakibaie et al., 2017; Vaigankar et al., 2018). However, the application of bacterial Bio-TeNPs might be limited due to their toxicity (Vaigankar et al., 2018). There are few reports on the application of fungal Bio-TeNPs. Moreover, the toxicity of fungal Bio-TeNPs has not been evaluated at present.

In this study, the high tellurite resistance fungus *Mortierella* sp. AB1 was isolated. It could reduce Te(IV) to produce Bio-TeNPs. Moreover, the nano-characteristics and antibacterial activity of the Bio-TeNPs were measured. Additionally, the antioxidant capacity and cytotoxicity of the Bio-TeNPs were evaluated.

## Materials and methods

### Materials

The strain AB1 was isolated in this study. Potassium tellurite (K_2_TeO_3_) was purchased from Shanghai Yien Chemical Technology (Shanghai, China) Co., Ltd. Strains *Shigella dysenteriae* CMCC 51252, *Escherichia coli* ATCC 25922, *Enterobacter sakazakii* ATCC 51329, and *Salmonella typhimurium* ATCC 14028 were obtained from the Strain Preservation Center of Hubei Centers for Disease Control and Prevention.

### Isolation and identification of strain *Mortierella* sp. AB1

Strain AB1 was isolated from the sewage sludge behind the pollutant analysis laboratory in Hubei Normal University. Isolation experiment of the strain according to our previous study (Wu et al., 2019). The obtained sample was diluted with 0.85% (w/v) sterile saline solution and spread on Luria-Bertani (LB) Agar plates containing 0.1 mmol/L K_2_TeO_3_. Then, the cultures were incubated at 37 °C for 16 h. Strain AB1 was separated and purified from these plates. The purified cultures were further experimented at 28°C with Potato Dextrose Agar (PDA).

For identification of strain AB1, DNA extraction was performed and ITS regions were PCR amplified by using the primers ITS1 (5′-TCCGTAGGTGAACCTGCGG-3′) and ITS4 (5′-TCCTCCGCTTATTGATATGC-3′). The TSINGKE DNA Gel Recovery Kit (Code No. GE0101) was used to obtain the target fragments. Sequence similarity searches were performed in the NCBI (National Center for Biotechnology Information) database. Then, a neighbor-joining tree was constructed using MEGA 11.0 and evaluated using 1,000 bootstrap replications based on the similarity sequences.

### Determination of tellurite tolerability and reducibility of strain AB1

The test to determine the Te(IV) tolerability and reducibility of strain AB1 according to the literature with some modifications (Wu et al., 2019; Calvillo-Medina et al., 2020). Briefly, corn meal medium (corn meal 40 g/L, KNO_3_ 2 g/L, NaH_2_PO_4_ 1 g/L, and MgSO_4_⋅7H_2_O 0.3 g/L, and agar 20 g/L) was used to determine the Te(IV) tolerability of *Mortierella* sp. AB1. Strain AB1 was inoculated on corn meal agar plates with various concentrations of Te(IV) (0∼25 mmol/L) at 28°C for 7 days.

Furthermore, modified potato dextrose broth (PDB) medium (potato 200 g/L, glucose 20 g/L, KNO_3_ 2 g/L, NaH_2_PO_4_ 1 g/L, and MgSO_4_⋅7 H_2_O 0.3 g/L) was used to measure the Te(IV) reducibility of strain AB1. Firstly, the hyphae of strain AB1 were inoculated into modified PDB medium in a shaker (180 r/min) for 2∼3 days at 28°C as seed fermentation broth. Then, a 5% (v/v) seed inoculum was inoculated into modified PDB medium with 0.5 mmol/L K_2_TeO_3_ and incubated at 28°C. To determine the Te(IV) residue in the medium, 500 μL culture medium was harvested and centrifuged at 12,000 rpm for 10 min to obtain the supernatants every 24 h. The residual concentration of Te(IV) was measured by a multifunctional microplate reader (SpectraMax i3x, Molecular Devices, CA, USA) based on the NaBH_4_ method (Molina et al., 2010).

### Synthesis and extraction of the biogenetic Te(0) nanoparticles

The seed inoculum described in 2.3 was inoculated into 100 mL modified PDB medium with 0.5 mmol/L K_2_TeO_3_ and incubated in a shaker (180 r/min) at 28°C for 7 days. Mycelia were collected by suction filtration. Then the obtained mycelia were milled in liquid nitrogen and collected with ddH_2_O. Next, the suspension was centrifuged at 10,000 rpm for 10 min and washed thrice with ddH_2_O. Further, the former products were washed three times with 1.5 M Tris-HCl buffer (pH 8.3) containing 1% (w/v) SDS and washed twice with ddH_2_O (Wu et al., 2019). Bio-TeNPs were further purified using an n-octyl alcohol-water partitioning system (Shakibaie et al., 2010). At last, the tellurium nanoparticles were washed and resuspended in ddH_2_O (10 mL). To determine the concentration of the Bio-TeNPs, the obtained materials were dried to constant weight at 80°C.

### Characterization of biogenetic Te(0) nanoparticles

To understand the morphology and composition of Bio-TeNPs, the obtained nanoparticles as described in section “Synthesis and Extraction of the Biogenetic Te(0) Nanoparticles” were further analyzed. Transmission Electron Microscopy and element mapping were performed on an FEI Tecnai G2 F30. Fourier Transform Infrared Spectroscopy and X-ray photoelectron spectroscopy analyses were carried out as in our previous study (Xia et al., 2018).

### Antibacterial ability tests

The inhibition zone tests were carried out for antibacterial ability evaluation. *S*. *dysenteriae*, *E*. *coli*, *E*. *sakazakii*, and *S*. *typhimurium* were cultured on Mueller Hinton Agar (MHA) media at 37^°^C overnight. Then, the cultured bacteria were collected from the MHA plates. The collected bacteria were washed and resuspended in 0.85% (w/v) saline solution and then diluted to turbidity 0.5 (∼1 × 10^8^ cfu/mL) using 0.85% (w/v) saline solution by a turbidity meter (Vitek 2 DensiCHEK). These four pathogen suspensions were plated on MHA plates. Each plate was coated with 50 μL of bacterial suspension. Next, one sterilized Oxford cup was placed on each plate. Different concentrations (0.1, 1, or 10 mg/mL) of Bio-TeNPs (100 μL) were added to sterilized Oxford cups. Subsequently, the plates were cultured at 37°C for 15 h. The diameters of the inhibition zones on the plates were recorded.

Additionally, a growth curve was used to evaluate the antibacterial ability of Bio-TeNPs. The growth conditions of *E*. *coli* and *S*. *typhimurium* in Mueller Hinton Broth (MHB) media were detected under 0 0.1, 1, and 10 mg/mL Bio-TeNPs. Then, 200 μL cultures were taken every 2 h and measured by a microplate reader (SpectraMax i3x, Molecular Devices, CA, USA) at 670 nm. Meanwhile, MHB medium and MHB medium with 1 mg/mL Bio-TeNPs were used as blanks for the control and experimental groups, respectively.

### Live/dead cell staining

A live/dead cell staining experiment was carried out to observe the bactericidal effect of Bio-TeNPs. The overnight cultured *E*. *coli* and *S*. *typhimurium* were collected and resuspended on MHB media (turbidity 0.5∼1 × 10^8^ cfu/mL). Then, they were cultured under 0 or 1 mg/mL Bio-TeNPs in a shaker (150 rpm) at 37°C for 4 h. Next, the cells were collected and washed twice with 0.85% (w/v) saline solution. The obtained cells were stained by the LIVE/DEAD Bacterial Staining Kit (BBcellProbe^®^ N01/PI, BestBio) according to the manufacturers’ instructions (Ren et al., 2020). The stained cells were observed by laser scanning confocal microscopy (LSCM, Nikon Eclipse Ti, Japan) after washing twice with 0.85% (w/v) saline solution.

### Morphological observation of microorganisms

Bacteria (∼1 × 10^8^ cfu/mL) were cultured under 0 or 1 mg/mL of Bio-TeNPs in MHB at 150 rpm and 37°C for 4 h. Thus, 2 mL cultures were collected and treated as described in our previous study (Xia et al., 2016). The morphology of *E*. *coli* and *S*. *typhimurium* were observed by scanning electron microscope (SEM).

### Antioxidant activity test

The DPPH radical scavenging assay was performed to evaluate the antioxidant activity of Bio-TeNPs (Wu et al., 2020). Briefly, 3 mL of a methanolic solution of DPPH (0.05 mM) was mixed with 0.5 mL of the Bio-TeNPs dispersed in ddH_2_O at different concentrations (0.078∼1.25 mg/mL). Then, the resulting mixtures were incubated in dark at room temperature for 30 min and the absorbance of the resulted solutions were measured at 517 nm by a multifunctional microplate reader (SpectraMax i3x, Molecular Devices, CA, USA). The percentage of DPPH inhibition was calculated by using the following formula:


$$\mathrm{DPPH}\mathrm{inhibition}\,=\,\frac{\mathrm{Absorbance}\mathrm{of}\mathrm{control}-\mathrm{Absorbance}\mathrm{of}\mathrm{test}}{\mathrm{Absorbance}\mathrm{of}\mathrm{control}}\times 100\%$$

in which, absorbance of control (without samples, DPPH solution only), and absorbance of test (different concentration samples, DPPH solution). Each experiment was performed in triplicate repeats.

### Cell counting kit-8 assay for evaluation of biogenetic Te(0) nanoparticles cytotoxicity

In order to identify the cytotoxicity of Bio-TeNPs, the Cell Counting Kit-8 (CCK-8) experiment was carried out according to the manufacturer’s instructions (APExBIO, USA). Vero-E6 cells (1.0 × 10^4^ cells/100 μL per well) were cultured for 24 h at 37°C (5% CO_2_). Then, 0, 0.1, 1, and 10 mg/mL Bio-TeNPs were used to treat the prepared cells for 24 h, respectively. The culture medium was removed and washed twice with PBS, and then 100 μL fresh DMEM (Dulbecco’s modified Eagle’s medium) was added to each well. Next, 10 μL of CCK-8 were added to each wells and then incubated at 37°C for 3 h. The absorbance was measured by a multiplate reader at 450 nm.

## Results

### Isolation and identification of strain *Mortierella* sp. AB1

**FIGURE 1:**
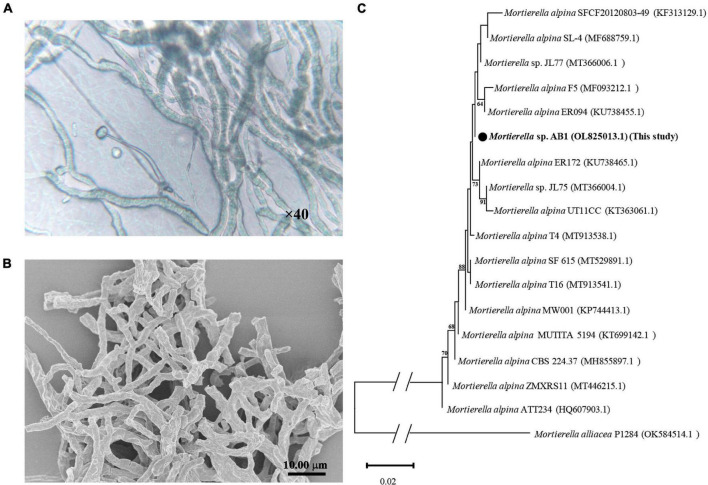
Morphological and phylogenetic analysis of *Mortierella* sp. AB1. **(A)** Images captured under a light microscope under 40× magnification. **(B)** Images captured under a scanning electron microscope. **(C)** Phylogenetic tree inferred from the Neighbor-Joining (NJ) analysis based on the ITS1-ITS4 sequence of strain *Mortierella* sp. AB1.

Strain AB1 was screened by LB agar plates containing Te(IV). The morphological and phylogenetic characteristics of the purified strain AB1 were then further analyzed. The strain was observed by light microscopy (×40) (Figure 1A) and SEM (Figure 1B). The results indicated that hyphae grew and branched to form a filamentous network, which is typical of molds. Moreover, the obtained gene sequence (OL 825013.1) showed high similarity to the *Mortierella* strains (99.18∼100.00%). The phylogenetic analysis indicated that strain AB1 clustered in the branch with other members of the *Mortierella* genus (Figure 1C). These results suggested that strain AB1 belonged to the *Mortierella* genus. Later, strain *Mortierella* sp. AB1 was deposited in the China Center for Type Culture Collection (CCTCC M 20211177).

### Tellurite tolerability and reducibility of *Mortierella* sp. AB1

**FIGURE 2:**
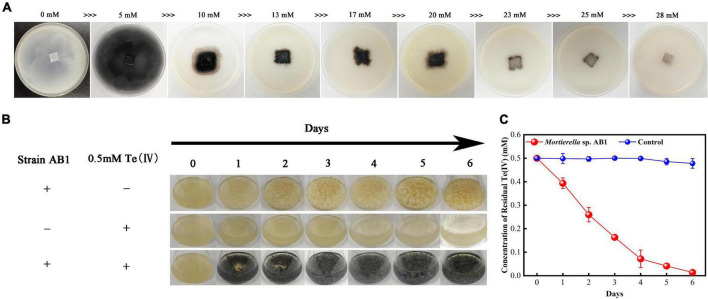
Te(IV) tolerance and reducibility test. **(A)** Strain growth on corn meal agar plates with different concentrations of Te(IV) (0∼28 mM). **(B)** Strains were grown in PDB medium with or without Te(IV). **(C)** The reduction curve of strain AB1 under 0.5 mmol/L Te(IV) in PDB medium, and 0.5 mmol/L Te(IV) in PDB medium without strain incubation was used as a control. Data are shown as the mean ± SD of three biological replicates.

Corn meal medium plates containing different concentrations of Te(IV) (0–30 mmol/L) were used to determine the Te(IV) tolerability of strain AB1. The results showed that the hyphae were white without Te(IV), while the hyphae were black with Te(IV) (Figure 2A). This result indicated that the black substance might be the Te(0) reduced by strain AB1. Because Te(0) was black color. The growth of mycelium on the plate was gradually restricted with increasing tellurium concentration (Figure 2A). When the concentration of Te(IV) was higher than 25 mmol/L, no obvious growth was observed on the plates after incubation for 7 days (Figure 2A). This result suggested that the minimal inhibitory concentration (MIC) of strain AB1 to Te(IV) was 25 mmol/L. Modified PDB medium containing 0.5 mmol/L Te(IV) was used to determine the Te(IV) reducibility of strain AB1. The mycelium was black in the bottle with Te(IV), which was similar to the phenomenon in the agar plates. The black color gradually deepened with the extension of time (Figure 2B). Meanwhile, the residual level of Te(IV) was decreased. No residual Te(IV) could be detected after 6 days of incubation (Figure 2C). These results implied that strain AB1 exhibited excellent Te(IV) resistance and reduction ability.

### Characteristics of biogenetic Te(0) nanoparticles produced by *Mortierella* sp. AB1

**FIGURE 3:**
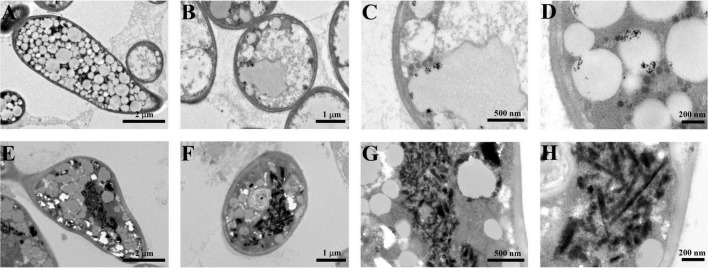
Bio-TeNPs observation in the cells of strain AB1. Strain AB1 was incubated with 0 mmol/L Te(IV) **(A–D)** and 0.5 mmol/L Te(IV) **(E–H)**, respectively. Then, the cells were collected to prepare ultrathin sections and observed under SEM.

**FIGURE 4:**
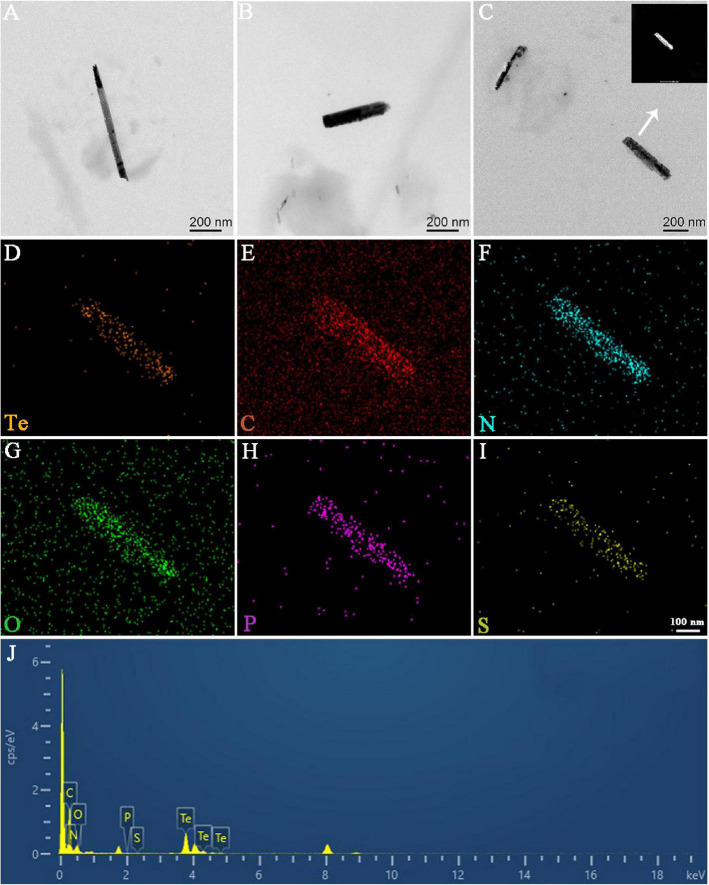
TEM-element mapping analysis of Bio-TeNPs. The morphology of Bio-TeNPs **(A–C)**. The Te **(D)**, C **(E)**, N **(F)**, O **(G)**, P **(H)**, and S **(I)** in Bio-TeNPs were analyzed by element mapping. The relative contents of each element were analyzed by EDS **(J)**.

In this study, Bio-TeNPs were synthesized by strain AB1. Strain AB1 was incubated with 0.5 mmol/L Te(IV) for 6 days. To confirm the production of Te nanostructures, ultrathin sectioning of cells and TEM were carried out to analyze the location of Bio-TeNPs in the cells. The TEM results showed numerous Te nanorods intracellularly when the strain was incubated with 0.5 mmol/L Te(IV) for 7 days (Figures 3E–H). In contrast, under the same culture conditions, no Te nanostructures were produced in the control cells without the addition of Te(IV) (Figures 3A–D). The Bio-TeNPs were extracted from cells of strain AB1. The components and morphology of Bio-TeNPs were analyzed by TEM-element mapping. The results showed that the Bio-TeNPs had diameters ranging from 100 to 500 nm and an irregular rod-like structure (Figures 4A–C). Elemental analysis indicated that Te was evenly distributed on the nanorods (Figure 4D). Meanwhile, C, N, O, P, and S elements were also found in the nanorods (Figures 4E–J). These results implied that fungal organics and Te were the main components of the Bio-TeNPs.

**FIGURE 5:**
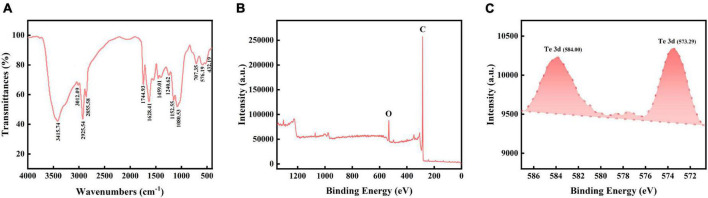
FT-IR and XPS analysis of Bio-TeNPs. **(A)** Bio-TeNPs consisted of proteins, lipids, aromatic compounds, and carbohydrates according to the FT-IR results. Survey spectrum of Bio-TeNPs with **(B)**, high resolution spectrum of Te3d **(C)** based on XPS analysis.

Moreover, the FTIR spectra presented peaks at 432.19 (S-S stretch), 576.19 (C-S stretch), 707.35 (C-S stretch), 1080.53 (S-H stretch), 1152.55 (CN stretch), 1240.62 (aryl-O stretch), 1459.01 (aromatic ring stretch), 1628.41 (C = C stretch), 1744.93 (alkyl carbonate), 2855.58 (methyne C-H stretch), 2925.54 (methyl C-H stretch), 3012.09 (olefinic group), and 3415.74 (hydroxyl group) cm^–1^ (Figure 5A; Coates, 2006). These results indicated that the Bio-TeNPs may contain proteins, lipids, aromatic compounds, and carbohydrates. The XPS total spectrum was used to analysis its composition and elemental valence state. The results also illustrated that the Bio-TeNPs were consisted of C and O element which might belong to organic substances in Bio-TeNPs (Figure 5B). This was consistent with the element mapping results. In addition, the XPS spectrum exhibited Te3d peaks at 573.5 and 583.9 eV, which were attributed to Te(0) (Figure 5C; Yang et al., 2012; Wu et al., 2019). This result indicated that the Te(IV) reduction product was Te(0) in strain AB1 and that Te(0) was the primary constituent of the Bio-TeNPs.

### Antibacterial activity of biogenetic Te(0) nanoparticles

**FIGURE 6:**
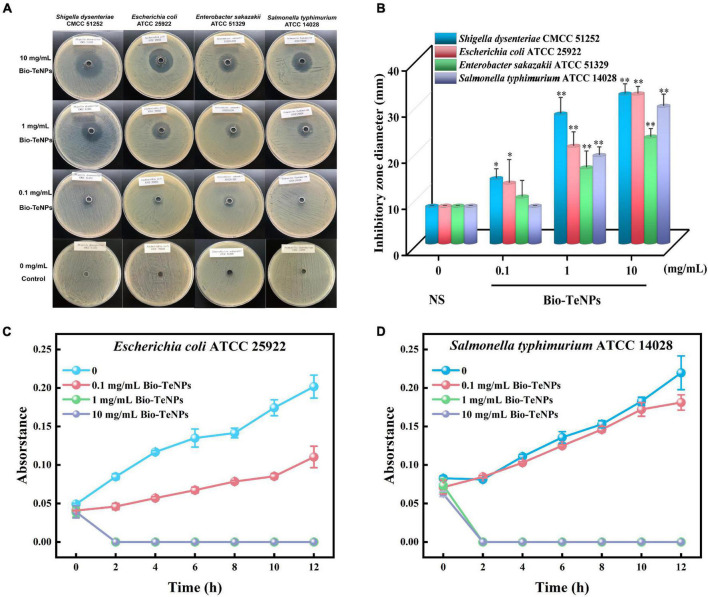
Antibacterial activity of Bio-TeNPs. **(A)** The inhibition zone images of 0.85% (w/v) normal saline (NS) and Bio-TeNPs (10, 1, and 0.1 mg/mL) on *S*. *dysenteriae*, *E*. *sakazakii*, *E*. *coli*, and *S*. *typhimurium*. **(B)** The inhibition zone diameter of NS and Bio-TeNPs (10, 1, and 0.1 mg/mL) on *S*. *dysenteriae*, *E*. *sakazakii*, *E*. *coli*, and *S*. *typhimurium*. **(C)** The growth curve of *E*. *coli* in MHB medium with or without 1 mg/mL Bio-TeNPs. **(D)** The growth curve of *S*. *typhimurium* in MHB medium with or without 1 mg/mL Bio-TeNPs. Data are shown as the mean ± SD (*n* = 3). Significance was analyzed with one-way ANOVA (*P < 0.05; ^**^P < 0.01).

Inhibition zone tests were carried out to test the antibacterial activity of the Bio-TeNPs. Noticeable inhibition zones were found in *S*. *dysenteriae*, *E*. *sakazakii*, *E*. *coli* and *S*. *typhimurium* plates after treatment with 10 and 1 mg/mL Bio-TeNPs (Figure 6A). The inhibition diameters of the Bio-TeNPs against *S*. *dysenteriae*, *E*. *sakazakii*, *E*. *coli* and *S*. *typhimurium* were 32.333 ± 1.202(10 mg/mL), 28.000 ± 2.000 (1 mg/mL), 14.000 ± 1.155 (0.1 mg/mL), 23.000 ± 1.000 (10 mg/mL), 16.333 ± 2.028 (1 mg/mL), 10.000 ± 2.000 (0.1 mg/mL), 32.333 ± 0.882 (10 mg/mL), 21.000 ± 1.732 (1 mg/mL), 13.000 ± 2.887 (0.1 mg/mL), 29.667 ± 1.453 (10 mg/mL), 19.000 ± 1.000 (1 mg/mL), and 8.000 ± 00.000 (0.1 mg/mL) mm, respectively. The inhibition diameters of the control group against these four kinds of pathogens were 8.00 ± 0.00 mm, as was the outer diameter of the Oxford Cup (Figures 6A,B). The inhibition diameter was positively correlated with the concentration of the Bio-TeNPs. This result indicated that these four kinds of pathogens were sensitive to Bio-TeNPs.

**FIGURE 7:**
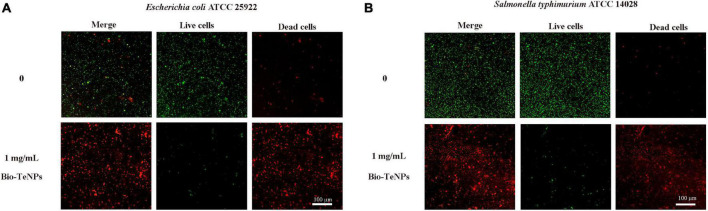
Live/dead cells analyzed by LSCM. **(A)** Fluorescence images of *E*. *coli* with or without 1 mg/mL Bio-TeNPs for 4 h. **(B)** Fluorescence images of *S*. *typhimurium* with or without 1 mg/mL Bio-TeNPs for 4 h. Most of the cells died after 4 h of treatment with Bio-TeNPs. Live/dead staining is shown as green for live cells and red for dead cells.

Furthermore, the antibacterial ability of the Bio-TeNPs were also detected by a growth experiment in MHB medium. *E*. *coli* and *S*. *typhimurium* were selected for further analysis. The results showed that the absorbance of *E*. *coli* and *S*. *typhimurium* was significantly inhibited by 1 and 10 mg/mL Bio-TeNPs compared to the control group (Figures 6C,D). This was supported by the results of live/dead cell staining. The proportion of live cells of *E*. *coli* and *S*. *typhimurium* was significantly decreased after treatment with 1 mg/mL Bio-TeNPs, while the proportion of dead cells of *E*. *coli* and *S*. *typhimurium* was significantly increased compared to those without Bio-TeNPs treatment (Figures 7A,B). All results revealed that the Bio-TeNPs exhibited excellent antibacterial ability.

### Morphological changes of pathogens after treatment with biogenetic Te(0) nanoparticles

**FIGURE 8:**
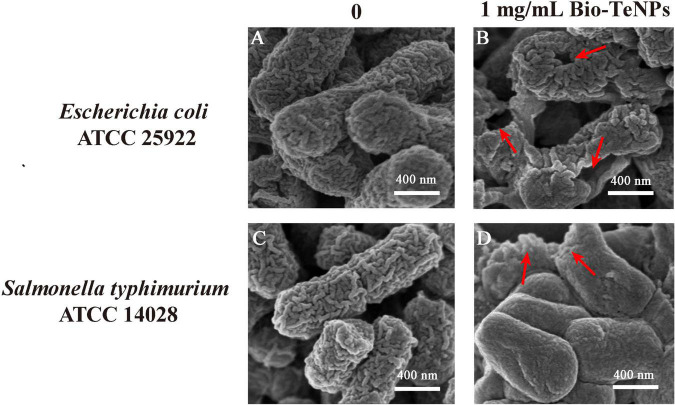
Morphological observation of *E*. *coli* and *S*. *typhimurium* cells. SEM image of *E*. *coli* with 0 **(A)** and 1 mg/mL Bio-TeNPs **(B)**. SEM image of *S*. *typhimurium* with 0 **(C)** and 1 mg/mL Bio-TeNPs **(D)**.

The morphology of *E*. *coli* and *S*. *typhimurium* was observed by SEM with or without Bio-TeNPs. *E*. *coli* and *S*. *typhimurium* cells were intact in the control group (Figures 8A,C). However, the *E*. *coli* cells were obviously broken (Figure 8B), and *S*. *typhimurium* cells were shriveled (Figure 8D) after treatment with 1 mg/mL Bio-TeNPs for 4 h. These results suggested that Bio-TeNPs can destroy *E*. *coli* and *S*. *typhimurium*.

### Antioxidant activity of biogenetic Te(0) nanoparticles

**FIGURE 9:**
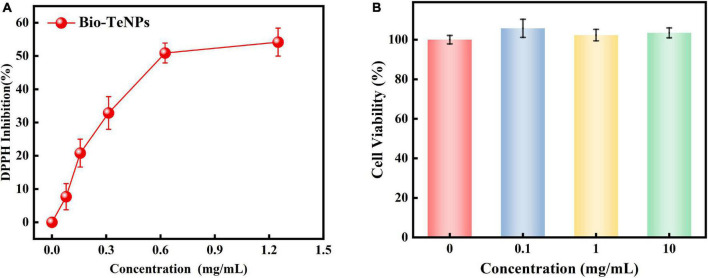
Antioxidant activity **(A)** and cytotoxicity **(B)** of Bio-TeNPs at different concentrations. Data are shown as the mean ± SD of three biological replicates.

The DPPH radical scavenging power assay was used to assess Bio-TeNPs’ capacity of antioxidant activity. The DPPH inhibition rate of Bio-TeNPs were evaluated at different concentrations. The results showed that the concentration of Bio-TeNPs positively correlated with the inhibition rate. The inhibition also was visually identified color change from violet to yellow indicates. Moreover, the maximum DPPH inhibition rate of Bio-TeNPs was 54% (Figure 9A). It suggested that Bio-TeNPs showed excellent antioxidant capacity.

### Cytotoxicity of biogenetic Te(0) nanoparticles

The cytotoxic activity of the Bio-TeNPs were analyzed by the CCK8 test. The results revealed out that the cell viability with Bio-TeNPs treatment at 0, 0.1, 1, and 10 mg/mL exhibited no significant difference (Figure 9B). It suggested that Bio-TeNPs showed no toxic to Vero-E6 cells.

## Discussion

Fungi play a major role in the bioremediation of various environmental pollutants owing to their robust morphology and diverse metabolic capacity (Deshmukh et al., 2016). Previous microbial Te(IV) bioremediation studies have mainly focused on bacteria (Vavrova et al., 2021), and fungal Te(IV) reduction studies have primarily concentrated on Te(0) nanoparticle production (Espinosa-Ortiz et al., 2017; Abo Elsoud et al., 2018; Barabadi et al., 2018; Liang et al., 2019, 2020). In this study, we found that the fungus *Mortierella* sp. AB1 could resist to 25 mmol/L Te(IV), which was higher than the Te(IV) resistance reported previously in bacteria and archaea (Maltman and Yurkov, 2019). Currently, there is a lack of Te(IV) resistance data of other reported Te(IV)-resistant fungi (Espinosa-Ortiz et al., 2017; Abo Elsoud et al., 2018; Barabadi et al., 2018; Liang et al., 2019, 2020).

Moreover, 0.5 mmol/L Te(IV) was removed by strain AB1 in 6 days. The Te(IV) reduction efficiency of strain AB1 and other fungi (Liang et al., 2019) was lower than that in bacteria (Wu et al., 2019). This might due to the growth velocity and related biomass were lower than those in bacteria under laboratory conditions (Wang et al., 2010). However, fungal bioremediation might possess advantages over bacterial bioremediation in the environment of intended application; these advantages include high Te(IV) resistance, growth in extreme and fluctuating environments and formation of mycelial networks to enhance remediation ability (Medaura et al., 2021). Accordingly, strain AB1 exhibited great potential in Te(IV) contamination bioremediation.

Generally, the microbial Te(IV) reduction activity is through the production of Bio-TeNPs (Zambonino et al., 2021). In *P*. *chrysosporium* (Espinosa-Ortiz et al., 2017) and *Shinella* sp. WSJ-2 (Wu et al., 2019), Bio-TeNPs were deposited in the intracellular, which was similar to strain AB1 in this study. It indicated that the Te(IV) reduction might occur in the intracellular among these microbes. Bio-TeNPs have also been observed on the surface of microorganisms or in media for some species, such as *A*. *pullulans, M*. *humilis, T*. *harzianum*, and *P*. *glomerata* (Liang et al., 2019, 2020). Presence of extracellular Bio-TeNPs might be due to extracellular Te(IV) reduction and release from broken cells. Bio-TeNPs exist in different shapes, such as granular (Abo Elsoud et al., 2018; Barabadi et al., 2018; Liang et al., 2019), pillar and needle shapes (Liang et al., 2019; Wu et al., 2019). The nanorods produced by strain AB1 were typical needles. The different shapes might be due to the different constituents in Bio-TeNPs. All Bio-TeNPs consist of Te(0) and organics. However, the organics from different microorganisms might be different. Accordingly, the shapes and even the applications of Bio-TeNPs in various microbes are diverse due to the different organics.

Additionally, the Bio-TeNPs produced by strain AB1 showed excellent antibacterial ability against *S*. *dysenteriae*, *E*. *sakazakii*, *E*. *coli*, and *S*. *typhimurium*, and antioxidant properties in this study. Currently, many bacterial Bio-TeNPs have exhibited potential in antibacterial applications (Zambonino et al., 2021). Cytotoxicity was observed in Bio-TeNPs produced by *Shewanella baltica* (Vaigankar et al., 2018). Consequently, the toxicity might limit the application of some bacterial Bio-TeNPs. However, Bio-TeNPs generated by strain AB1 showed no cytotoxicity in this study. This result suggested that Bio-TeNPs of strain AB1 might be more useful than some bacterial Bio-TeNPs in consideration of toxicity. Furthermore, the antibacterial ability of fungal Bio-TeNPs is currently unknown. Only the Bio-TeNPs produced by *A. welwitschiae* were found to have antibacterial activity against *E. coli* and *Staphylococcus aureus* (Abo Elsoud et al., 2018). One of the important advantages of antibacterial nanomedicines is their potential to substitute for antibiotics. However, the *A. welwitschiae* strain is a promising antibiotic-producing strain (Omeike et al., 2019; Maliehe et al., 2022). This means that the antibacterial activity of Bio-TeNPs produced by *A. welwitschiae* might be due to its antibiotics. Consequently, the Bio-TeNPs produced by strain AB1 were better in antibacterial application than those produced by *A. welwitschiae*. In previous study, the *Mortierella* genus strain, *M. humilis* also showed the Bio-TeNPs synthesis ability (Liang et al., 2019). However, the medium for producing Bio-TeNPs by *M. humilis* was more expensive than that of strain AB1, and the yield of Bio-TeNPs were lower than that of strain AB1 in this study. Besides, the potential application and cytotoxicity of Bio-TeNPs produced by *M. humilis* were not detected. Accordingly, strain AB1 showed greater application potential than *M. humilis*.

## Conclusion

In this study, a high Te(IV) tolerance fungus was isolated and identified as *Mortierella* sp. AB1, which was also with the ability to reduce highly toxic Te(IV) to less toxic Te(0). The reduced Te(0) formed Bio-TeNPs in the intracellular of strain AB1. The synthesized Bio-TeNPs ranged from 100 to 500 nm and consisted of Te(0), proteins, lipids, aromatic compounds, and carbohydrates. Moreover, the Bio-TeNPs exhibited excellent antibacterial activity against *S*. *dysenteriae*, *E*. *coli*, *E*. *sakazakii*, and *S*. *typhimurium* according to inhibition zone tests. This was further supported by the results of growth curve experiments, live /dead cell staining and cell morphology observation. Additionally, the obtained Bio-TeNPs exhibited excellent antioxidant capacity without cytotoxicity. The strain AB1 and it produced Bio-TeNPs showed great potential in bioremediation and antibacterial and antioxidant application.

## Data availability statement

The original contributions presented in the study are included in the article/supplementary material, further inquiries can be directed to the corresponding author/s.

## Author contributions

BA: investigation, data curation, and formal analysis. FH: investigation, methodology, data curation, and validation. JL: investigation and project administration. JT: funding acquisition, conceptualization, and supervision. ZT: data curation and formal analysis. HJ: funding acquisition and supervision. XS: project administration and writing—review and editing. JJL: resources and writing—review and editing. JH: funding and supervision. YH: writing. XX: writing, conceptualization, supervision, and funding acquisition. All authors contributed to the article and approved the submitted version.
